# Stakeholder-engaged development of a rapid test for detection of acute HIV infection

**DOI:** 10.21203/rs.3.rs-4243639/v1

**Published:** 2024-04-19

**Authors:** Natalia M. Rodriguez, Lara Balian, Ishita Kataki, Cealia Tolliver, Julio Rivera-De Jesus, Jacqueline C. Linnes

**Affiliations:** Purdue University; Purdue University; Purdue University; Purdue University; Purdue University; Purdue University

**Keywords:** HIV diagnostics, Acute HIV infection, rapid testing, human-centered design, stakeholder engagement, implementation

## Abstract

**Background/Objective:**

The utilization of rapid HIV tests has been effective at reducing transmission rates in high-risk populations by allowing individuals to receive diagnosis in as little as one minute and begin treatment. However, no current rapid tests can detect HIV immediately after infection in the acute HIV infection (AHI) phase, when the virus is at its most infectious, and instead require a waiting period of up to 90 days after exposure. Rapid HIV tests to detect AHI are currently under development. Investigation of stakeholder perspectives and context-specific needs are critical to ensure successful translation of novel AHI tests. The objectives of this study were to 1) understand context-specific factors such as barriers to HIV testing in Indiana, a state with one of 48 prioritized counties for HIV elimination; 2) assess the acceptability of a novel rapid AHI test, and 3) identify key implementation considerations for such a device, including ideal end-users.

**Methods:**

Semi-structured in-depth interviews were conducted with staff (n = 14) and clients (n = 5) of Indiana-based organizations that conduct HIV testing, including syringe service programs. Utilizing human-centered design frameworks, interview guides were developed and tailored to each participant group to understand their experiences with HIV testing, perspectives on a novel rapid AHI test in development, and preferences for self-testing versus testing by a community health worker (CHW) or a peer recovery coach. Thematic analysis was conducted to identify major themes, including barriers to HIV testing and perceived benefits and concerns of the proposed AHI test.

**Results:**

Overall acceptability for a novel AHI rapid test was high with a greater preference for CHW/Peerled testing. While self-testing was not a preferred modality, it was still seen as a potential tool to reach and address key barriers among high-risk individuals. Key considerations for implementation emphasized accuracy, cost-effectiveness, ease of use, ensuring access to counseling, education, and navigation to care while maintaining a human element to self-testing.

**Conclusion:**

Stakeholder engagement is meaningfully informing the design, development, and implementation of rapid AHI testing in order to facilitate adoption among populations at high-risk for HIV.

## Background

The persistent burden of HIV (39 million worldwide [1] and 1.2 million in the U.S. [2]), particularly among marginalized populations, along with suboptimal testing rates, highlights the need to expand and innovate HIV testing efforts. HIV disproportionately affects sub-populations in the U.S., including people who inject drugs (PWID), who account for 1 in 15 HIV diagnoses, and who are considered high-risk due to sharing needles, syringes, or other drug injection equipment [3]. Further, despite some success in reducing transmission among PWID through syringe service programs, the expanding injection drug epidemic and effects of COVID-related reduction in healthcare access have led to HIV outbreaks among PWID, who are already marginalized from the mainstream HIV care continuum [4, 5]. The higher prevalence of HIV compounded with a range of social, economic, and demographic factors (e.g., stigma, education, income, rurality) can further increase risk for transmission, affect access to testing and care services, and potentially lead to worse outcomes and quality of life after HIV diagnosis in these high-risk populations [6].

HIV testing has long served as a preventive strategy for HIV, as people who know their status can make informed decisions about behaviors associated with HIV transmission, and those who test positive can engage in HIV treatment and reduce viral load to untransmissible levels. Yet, almost 40% of new HIV infections in the U.S. are transmitted from people who did not know they were infected, and 13% (158,500) of people don’t know their status that should be tested [7]. While those who reported at least one high-risk behavior for HIV (e.g. injecting drugs, unprotected sex) had higher rates of testing than those who did not (65.2% compared to 44.2% in 2016), 34.8% still had never been tested, and 65.8% had not been tested in the past 12 months [8].

The highest risk of HIV transmission occurs in the acute HIV infection (AHI) phase, the first stage of infection that occurs in the first 2–4 weeks [9]. Existing tests that are most used to screen for HIV are rapid antibody or antibody/antigen tests that can produce results within 1–30 minutes from fingerstick blood or oral fluid. These tests require a waiting period of up to 90 days after exposure as they cannot detect the virus at the AHI stage when infected individuals are the mostly likely to spread HIV to others. While nucleic acid tests can detect HIV earlier (10–33 days after exposure), these laboratory-based tests require intravenous blood draw, expensive equipment, time-consuming protocols, cold-chain reagent storage and trained personnel. As such, researchers are working to further advance rapid HIV testing to detect the virus at the acute phase [10–15].

Dr. Linnes and colleagues are developing a point-of-care (POC) AHI test capable of detecting the equivalent of < 1000 HIV virions/mL from a finger prick blood sample within 30 minutes. This test, called MicroRAAD, is a fully integrated, handheld, sample-to-answer device that uses reverse-transcription loop mediated isothermal amplification (RT-LAMP) to amplify HIV-1 RNA and provides a colorimetric readout for the amplification products via a lateral flow immunoassay. This platform can detect the virus within 90 minutes [11]. One of the most novel aspects of this device is achieving detection through whole blood sampling by automatically performing the multistep processes of isolating the virus from the sample, amplifying the RNA, and then transferring RT-LAMP products to the detection zone; making this a straightforward molecular testing platform for AHI. The test is designed to empower those at high-risk for HIV to detect and monitor their HIV status earlier following potential exposures.

This new testing paradigm has the potential to transform the HIV care continuum by making critical AHI detection possible at sites ranging from substance abuse treatment facilities to syringe services, addressing access barriers in areas of high HIV risk. However, without ensuring that a diagnostic POC technology is linking patients into the existing care continuum, even the most exquisitely sensitive POC devices will fail to make a difference in clinical outcomes. The success of this, and all technology platforms, will depend on the extent of adoption by, and delivery to, end-users. According to human-centered design frameworks, this requires examination of context-specific needs early and throughout the design process with end-users and experts who have lived experience or engage with the priority communities of an innovation [16].

The Ending the HIV Epidemic initiative in the U.S. aims to reduce new incidences of HIV by 90% by 2030 and to “diagnose all people living with HIV as early as possible”.[17] Marion County, Indiana represents one of the 48 priority counties that account for more than half of all new HIV diagnoses in the U.S [18]. While testing of individuals at high risk for HIV varies by rurality (18.4% in rural, and 29% in urban areas in states with high HIV incidence) [19], the overall availability and usage of HIV related services among PWID and other marginalized populations are understudied in non-urban areas [20]. Therefore, this study sought to engage key stakeholders from organizations across Indiana who conduct HIV testing with PWID and other high-risk populations, as well as clients of syringe services or HIV testing services, to inform the development and implementation of the novel rapid AHI test. The objectives of this study were to 1) understand context-specific factors such as current experience with and barriers to HIV testing in Indiana; 2) assess the acceptability of this novel AHI test (that can detect the acute phase of HIV but takes longer to produce results than current HIV rapid tests) in this context, and 3) identify the target end-user and related implementation considerations for the device.

## Methods

Semi-structured interviews were conducted via Zoom between September 2020 – December 2021 with staff of organizations that conduct HIV testing in Indiana and clients who receive services at HIV organizations and/or syringe service programs in Indiana.

Staff of organizations were recruited via snowball sampling. Organizations with publicly available email addresses were emailed about the study, instructed on how to participate if interested, and encouraged to pass along the opportunity to other colleagues and HIV service organizations. Any staff member of an HIV organization was eligible to participate if they oversaw or conducted HIV testing.

Clients were recruited through passive methods, given the potential vulnerability of this population. Flyers were posted at local organizations upon their approval, and included information about the study, gift card incentive, and researchers’ contact information so that they could get in touch with the study team only if they wished to participate. Those who reached out were eligible to participate if they were at least 18 years old and received services at syringe service programs or HIV testing organizations in Indiana. All participants were given the option to be interviewed in-person, over the phone, or via Zoom, as they preferred for up to 60 minutes.

Two interview guides were developed and tailored to each participant group (staff and clients) based on human-centered design frameworks that call to understand unique perspectives, experiences, and context-specific factors of users and stakeholders in order to foster successful and equitable adoption of health technologies.[16] Staff were asked questions about their current testing process, including challenges or barriers they faced, while clients were asked about their experiences with HIV testing, including barriers and accessibility to testing.

To glean insights on the intended end-user and setting for an AHI HIV rapid test, clients and staff were asked about their thoughts on self-testing, testing by a community health worker (CHW), a frontline public health worker with a close understanding of the community served who acts as liaison between the community and health/social services [21], or a peer recovery coach, a person who has been successful in recovery from addiction or mental health issues and assists others in their recovery process [22]. Clients were asked how their behaviors would change if they knew they were positive or negative, and what they would do if they received a positive result. Similarly, organizations were asked about their thoughts on health behavior change among clients who learn their HIV status.

Lastly, both clients and staff were informed about the AHI test in development and that the goal of the test is to detect HIV a month earlier than existing rapid tests but the test would take longer (around 60 minutes) to produce test results. Participants were then asked, “Do you think this trade-off would be worthwhile to clients or providers?” and “Do you think there is a need to be able to detect HIV earlier for PWID and other at-risk populations?” Questions from the interview guides are provided in Table 1.

Interviews were conducted by trained study team members and audio-recorded and transcribed verbatim using Otter.ai. Pseudonyms were used for all participants. All transcripts were reviewed and edited independently by two study team members and reviewed by the interviewer if necessary to address any discrepancies or questions by the transcribers. When transcription was finalized, audio recordings were deleted to protect the identity of clients.

A codebook for each participant group was developed under the guidance of the principal investigator (PI) using inductive and deductive methods based on the interview guide, research questions, and preliminary transcript review. Each interview was independently coded in NVivo, a qualitative coding software, by two study team members trained in qualitative coding methods. The two coders met to reach consensus for how each interview was coded. Any remaining coding discrepancies were brought to the large study team and PI to reach agreement. Upon agreement and completion of all coding, two study team members per research objective led the analysis to identify themes, relationships, and patterns between participant groups and across all interviews which was then reviewed and finalized with the remaining study team.

This study was approved by Purdue University’s Institutional Review Board (IRB-2021–1437).

## Results

A total of 19 interviews were conducted (n = 5 clients, 14 staff). Of the five clients interviewed, two identified as male, two as female, and one unreported. Most identified as White (4) and one person as Hispanic. Two clients were experiencing homelessness and almost all had used intravenous drugs (4). Those who injected drugs reported sharing needles never (2), rarely (1), and half the time (1). The number of sexual partners in the past year ranged from 1–3. All clients knew their current partner’s HIV status and reported low condom use (3 never used condoms and 2 rarely used them).

Of the 14 staff interviewed, five were directors or managers of Testing and Outreach units in their organization, while the remaining 10 conducted HIV and/or sexually transmitted infection (STI) testing. Most of the people who performed HIV testing also provided or coordinated counseling and outreach services, and two identified as a Peer Recovery Specialist or CHW.

The staff interviewed represented 10 organizations in Indiana that provide HIV testing services. Most organizations also provided education and outreach on HIV and STIs, ranging from positing flyers in public spaces to actively going out to the community with testing kits, as well as healthcare navigation, and medical and non-medical case management (e.g. housing assistance). About half of the organizations also had their own HIV treatment and PrEP services. Some organizations were part of larger networks and partnerships, serving up to 45 counties and partnering with up to 75 other HIV/AIDS organizations. While few organizations were specifically focused on PWID and had a syringe service or harm reduction program, all organizations had experiencing working with high-risk populations and many targeted specific efforts to further reach populations in their area at high-risk for HIV or who have low utilization of HIV testing services.

### Experiences and Barriers to HIV Testing

**Figure 1:**
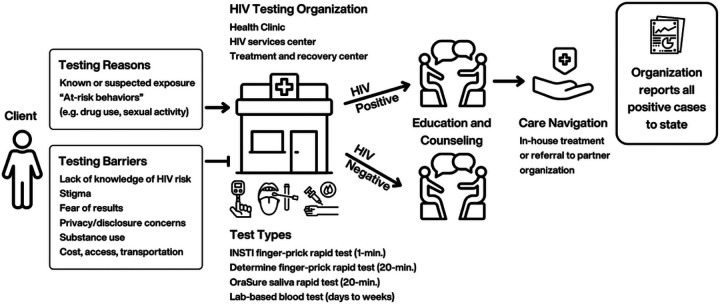
HIV Testing Experience Pathway

Figure 1 visually summarizes the HIV testing experience as described by interviewees. Clients come to organizations to get tested for a variety of reasons. From the organization perspective, a client may come once because of a known or suspected exposure whereas others come in regularly, anywhere from every 3 months to yearly, likely because of risky behaviors. Clients shared that choosing to get tested depended on their individual risk assessment such as drug use and sexual activity. Those who viewed their risk as high (e.g., due to injection drugs) tended to test more regularly and frequently. Clients reported accessing testing at treatment and recovery centers (e.g., needle exchange programs), HIV testing organizations, and/or doctor’s offices and were last tested anywhere from 1–3 months prior to their interview, either by a rapid test or lab-based blood test. All staff reported using rapid HIV testing as the initial test for clients. INSTI^®^ HIV-1/HIV-2 Antibody Test by bioLytical Laboratories Inc. [23] and Determine^™^ HIV-1/2 Ag/Ab by Abbott [24] were specifically mentioned as initial rapid tests that detect HIV from a finger prick of blood and yield results in one-minute and 20 minutes, respectively. OraSure, another rapid antibody test that detects HIV from oral fluid and yields results in 20 minutes, is used by staff as a second rapid test to confirm only positive INSTI results. Clients who test positive using a rapid test would then take a lab-based test that can take up to a week for results. The positive lab result officially confirms the HIV case so that the organization can report it to the Indiana State Health Department, per state law.

Many staff connect clients who test positive with a case manager to link directly to care within their organization, often starting them on treatment that day. Those who do not have treatment services within their organization, have strong partnerships with other organizations that they refer clients to. Many also noted that providing education before, during, and after testing is considered crucial for both positive and negative tests.

While most clients interviewed found HIV testing to be easy to access, staff mentioned key access barriers including many people not knowing where or how to access testing, concerns about cost, and transportation, specifically for high-risk populations such as PWID, people experiencing homelessness, or people who are incarcerated.

Stigma was viewed as one of the most significant barriers to testing, for clients, in terms of drug use, fear of results, and worrying about other people seeing results. From the staff perspective, stigma as a testing barrier is related to societal attitudes and beliefs about drug use and sex, and something that they hear from clients and are actively trying to improve through education and counseling, “*I think stigma is a huge reason, obviously, that people stay away from getting tested. They don’t want to feel dirty. They don’t want to be judged for their sexual activities” (James, HIV and STI Tester)*. Stigma can also be associated with lack of knowledge about HIV and resulting misconceptions about HIV risk, “Everybody still thinks this is mainly men versus men” (*Ana, Medical Assistant*), or creating fear of getting tested because others might find out *they’re afraid, you know, like, in a small town, that somebody might find out that they’re being tested or what if they are positive, then what are they going to do? People are just afraid, a lot of times to find out* (*Bonnie, HIV Tester/Counselor*).

### Acceptability of AHI rapid test

Acceptability of the described AHI rapid test was high. All participants who were asked if there was a need to detect HIV earlier in high-risk populations said yes. All clients and all but one staff who were asked if having a test that “detects HIV a month earlier than other tests but takes longer to receive results (around an hour instead of 15 minutes)” is a worth-while trade-off, agreed,

“An hour’s not too bad. It would be torture, it would be an hour of torture, but yeah, that’s pretty good… that’s impressive. It’s worth it, worth the hour of torture [laughs] … to have that opportunity”

(Terrence, client)

“I think it would be beneficial to the clients because when you tell them that they have to wait 90 days to find out from a risk that happened today. [Even] If that got jumped up to 60 days versus 90 days that’s going to help that person.”

(Carissa, HIV and STI Tester)

The potential for this test to reduce and prevent HIV treatment and facilitate early treatment for those who test positive, outweighed the few concerns, expressed by just two participants (both staff), Table 2.

### Target End-users

Possible end-users discussed for this test were: 1) client (i.e., self-testing), and 2) testing by a CHW or peer recovery specialist (i.e., CHW/peer-based testing). Table 3 outlines the benefits and limitations of each end-user as perceived by participants.

Overall, there was a greater preference for CHW/peer-based testing over self-testing, however clients were more comfortable and open to self-testing than staff. Despite staff hesitancy, most recognized that self-testing has “its place” and could serve as “a good tool for people to have”, or at the very least can be a last resort to reaching others who wouldn’t otherwise get tested, “*I think it could be dangerous, but I also see it as a way that maybe someone would have never received that test and they never would have got the nerve…so it’s definitely a tool. And I would love to have that tool to give somebody if they weren’t comfortable in testing with me or testing at our center…” (Blaire, Director of Outreach and Testing)*

Participants suggested that conducting the HIV rapid test could be done by anyone who is adequately trained, including on follow-up and linkage to care, “*I think it’s a good idea for somebody who’s trained to do the test and training doesn’t necessarily mean they have to be a doctor or a nurse. It just means that they should be somebody who’s prepared to give the results, whatever the results may be…and who knows like if it were to turn out positive, then you would want somebody who knows how to get connected to care and all of that, but I couldn’t care less whether it’s a doctor or not. Just someone who knows what they’re doing” (Terrence, client)*. These skills are some of the benefits mentioned for CHW/peer-based testing in Table 3.

Additionally, organizations preferred CHW/peer-based testing, especially for high-risk or hard-to-reach communities like PWID,

“I definitely think either peer-led or community health testing like we do, I think would definitely benefit [PWID] … I mean for us, we’re able to test quite a few injection drug users just because again we’re going to where they’re at, and we have an education component with that… nobody wants to be tested in the beginning, but then as the educator is halfway through the education, 6 of the 10 now want to be tested. So I think that component is very important because again people think that ‘this can’t happen to me’ or ‘I just don’t want to know’.”

(Kennedy, Director of Outreach Services)

### Implementation Considerations

Given the benefits and concerns for self-testing and CHW/peer-based testing, key considerations for implementing an AHI test were identified in Table 4.

Some organizations have implemented a mail-delivered home-based self-testing program during the COVID-19 pandemic for existing rapid tests [25], and offer insights into how they have succeeded at some of these suggestions and how successful the program was been. For Andy, Outreach Coordinator and Tester, that meant well written instructions, “…*I’m for that [program]. Mostly because the instructions are written really really well. Because it’s written to I believe a fifth or sixth grade reading level, which is great. I’m all for it because that’s the people who often need it don’t often have a master’s degree or anything like that” (Andy, Outreach Coordinator). Anita, HIV Testing Department Manager, providing an online option is a priority to address some of her concerns for adopting a program like this, “I’ve been under some pressure to get a testing program going where we’re maybe like shipping kits out to people. I’m opposed to that. I think it’s a poor use of nonprofit funds to spend $8 - $10 shipping. You know, who one may or may not actually follow up with. So as I move forward with that, what I’ll be doing is configuring to have, you know, obviously a screening process before we could send a test to a person, but also setting up like a zoom link with them so that our tester could work on a laptop. And, you know, hopefully at least sometimes have that virtual connection with the person.” Evy, Prevention Team Lead, finds in her experience that while the program hasn’t been as popular as they thought it would have been, clients have wanted that human connection as well, and have chosen to test themselves while connected with staff remotely, “… what we try to do, is do some like risk assessment with the folks, with the person when we’re talking to them before we either send or deliver to the test, and offer to them if they want us to go through the test with them either over the phone or via zoom, that we can do that. And actually, by and large, that people have said yes to that, which we were also a little surprised because we were kind of like, ‘okay, if somebody has this test in their hands, and I mean they’re simple they’re really easy to use’… but people actually have wanted to follow up with us, which is nice”*.

## Discussion

This study aimed to understand the current state of rapid HIV testing among high-risk populations in Indiana, to assess the acceptability of a novel rapid AHI test in this given context, and to determine the target end-users and related implementation considerations for such a device. While clients typically have access to rapid HIV testing at various frequented sites such as syringe service programs and substance use recovery organizations, persistent barriers including cost, access, transportation, and stigma, particularly among PWID, remain key challenges. Furthermore, the requirement of existing rapid tests for clients to wait up to 90 days post suspected exposures to test, is a significant barrier to preventing transmission and facilitating access to care in these populations. To that end, the need to detect HIV earlier among high-risk groups was expressed by many participants, and a novel AHI rapid test that would allow for this, despite taking up to an hour to produce results, was deemed highly acceptable. These findings both support and inform the continued development of this novel device.

This work can inform the development and implementation of broader emerging AHI tests. Portable RNA tests for POC AHI detection have recently been developed but not yet implemented in the field [26–29]. Additionally, alternative antigen-antibody 4th generation POC have been developed to detect AHI at the POC. While these are similar to existing HIV rapid tests detecting antibodies and provide results in under 30 minutes, they are designed to also detect the p24 antigen that is present during AHI. Analytical testing in frozen serum samples has shown good performance of these 4th generation POC tests in overall HIV detection [30], however, both field and multi-laboratory trials have shown that the antigen detecting capabilities of these 4th generation POC tests were unsuccessful. [31, 32] With additional incubation or sample preparation steps that free complexed p24 antigen [33], these 4th generation tests may also be better able to detect AHI. The work here, demonstrates that these additional steps, may be worthwhile to integrate into antigen-antibody 4th generation POC tests despite the added time to result.

While the FDA has approved qualitative laboratory-based HIV RNA tests for diagnosis, current CDC testing algorithms only recommend using laboratory RNA tests after HIV antigen/antibody testing for confirmation or genotyping, or if the first tests are indeterminant [34]. RNA-based AHI tests tend to be more expensive than antibody/antigen counterparts [35]. Babigumira et al compared the cost effectiveness of detecting AHI via POC RNA-based testing compared to existing detection of chronic HIV infections in Kenya and found that offering POC RNA-based testing to outpatients who present with symptoms of AHI would be a cost-effective strategy [36]. Similar analyses for screening of high-risk individuals in lower-burden countries would also be important to understand potential costs for implementation effectiveness in the US.

To inform further design of this novel test, potential end-users and testing modalities were discussed with participants to gauge preferences for the target end-user (self-testing vs CHW/peer-led testing) and related implementation considerations. While participants expressed important benefits and concerns for both end-user groups, the majority felt that CHW/peer-led testing would better meet the needs of high-risk populations, particularly regarding the need for counseling and navigation to care. These findings supports a growing body of literature on the acceptability of both HIV community-based/peer-led and self-testing more broadly [37–43]. Several reviews have found that while interest in self-testing is high among vulnerable groups because of convenience and privacy, many are concerned about the reliability and accuracy of tests and post-test linkage to care and counseling [38–40]. These are many of the same concerns that existed when HIV antibody self-tests were first instituted [44]. Clear and simple instructions for self-tests, including on next steps for a positive result, were mentioned by participants as important implementation considerations. With these in mind, others have found that self-testers can reliably and accurately conduct rapid HIV testing compared to trained healthcare providers [45].

This study also contributes to literature on model approaches for appropriate technology development that meaningfully consider and incorporate contextual investigation early in the design process of novel health technologies [16]. The utilization of human-centered design frameworks and engagement of diverse stakeholders to inform the acceptability and preferences of HIV testing interventions are key strategies to understand and promote their successful implementation, uptake and adoption [46, 47]. Relatedly, a key limitation of this study is that recruitment of clients was passive and relied on flyers posted at organizations that conduct HIV testing, limiting our participant pool to those with current access to testing. Future work must reach medically-underserved communities that are not yet engaged in HIV testing or care, as well as broader stakeholders including local health departments and insurance companies to understand implementation considerations related to reporting and reimbursement.

Given the generally positive responses towards the goal of detecting AHI with POC technology. The work has informed key next steps in accuracy, simplicity to use, and cost. To increase accuracy, the limit of detection is being improved with new RNA assay designs. Further, the manufacturing costs are being reduced with streamlined device design that uses fewer parts and requires fewer steps for assembly. Lastly, an internal amplification control ensuring test validity and easily interpretable results has recently been developed for SARS-CoV-2 and will be adapted to the AHI detection. The original MicroRAAD was designed to have minimal user steps but did not include components to provide user feedback and confidence that the 60-minute test was running correctly during use. Ongoing iterations of MicroRAAD prototypes with various user feedback modes (e.g. LEDs, progress screen, clear instructions for use and results interpretation) are currently under development and will be evaluated in future usability studies among CHWs, Outreach Coordinators, and Peer Recovery Coaches. The implementation of early-stage feasibility and acceptability studies combined with responsive technology design iteration and usability testing among the intended users brings together best practices of human- and equity-centered design.

## Figures and Tables

**Table 1.** Interview Guide Questions

Staff Interview Questions	Client Inreniew Questions
Can you tell me about the people that you serve? About how many clients does your oiganization serve? What are some of the typical demographics of the communities you serve? What are some of the key barriers to accessing HIV testing and treatment that you typically see among your clients (or before they become your clients)? How do you test people for initial HIV diagnosis? What specific test do you use? How do you feel about the test? Any issues? What kind of sample do you take? *(Saliva? Blood? Fingerprick?)* Where do you test them? Who performs the test? How often do you test people? *(just see them once and never again? Or do you see them frequently and offer testing often?)* In an ideal world, how often should we test people? PWID specifically? What are your thoughts on self-testing? Peer-based testing? CHW-led testing? What possible problems could these solve? What possible problems could these cause? What about for PWID? Which testing modality do you think would work best? Do you think testing your clients/letting them know their HIV status changes their health behaviors in any way? Engineers are currently working on a rapid HIV test that could detect acute HIV infection at least a month earlier than existing tests, but it takes longer (around 60 minutes) Do you think this trade-off would be worthwhile to clients or providers? Do you think there is a need to be able to detect HIV earlier for PWID/other at-risk populations?	Have you ever been tested for HIV? When was the last time? Why did you decide to get tested? How often do you get tested? How often would you like to get tested ideally? What was the test like? Who did the test? (Doctor? CHW? Peer?) Finger-prick? Saliva? How long did it take to know results? Any follow-up? Is it easy to access HIV testing whenever you want it? What are the barriers? Have you ever heard of an HIV rapid test? In your opinion, who should perform the test? (probe: chw, nurse, doctor, shelter staff, needle exchange service staff) Where would you ideally want to take the test? (probe: street, slielter. site, other) Would you be willing to conduct your own test? Or prefer someone you know to do it for you? Have you ever heard of a peer recovery coach/certified recovery speeialist/community health worker? Would you like to be able to work with one? If yes: how might they be helpful to you? If no: why not? If a peer recovery coach bad HIV tests on them and could test you anytime, would that be useful to you? Would you seek them out for HIV testing? Why/why not? If you could test yourself for HIV, with a test kind of like a pregnancy test that you could get at any pharmacy… would that be useful to you? Would you like the ability to be able to test yourself anytime? Why/Why not? What are some benefits of being able to test yourself? What do you think are possible downsides? When or how often would you test yourself? Would your behaviors change if you knew your were positive/negative? Would you know what to do/where to go/who to call if you were positive? Engineers are currently working on a rapid HIV test that could detect HIV infection at least a month earlier than existing tests, but it would take longer (around 1hr instead of 15 minutes) Do you think there is even a need to be able to detect HIV earlier? Do you think this trade-off (takes longer but get results earlier) would be worthwhile to you? Why/why not?

**Table 2:** Benefits and Concerns of the AHI rapid test

Benefits	
Reduce/prevent HIV transmission	*“… these two or three month periods of time that it takes to find out [if you have HIV]. That’s a lot of life going on in that period of time. And if you want to try to let all the people know that you might have come in contact within those two to three months. That’s not easy… I think it’s [the AHI test] a great idea. For all kinds of reasons, not just that* **there’s less time to be infecting other people, there’s an easier chance of finding the one that infected you.** *You know, there’s a lot of good stuff going on there if the time period is shortened down.” - Terrence, client*
Facilitate early treatment, particularly for high-risk clients who are sometimes lost to follow-up	*“…if they tell me it’s been three or four weeks ago, since they had the unprotected sex… my biggest worry is: are they really going to come back in 90 days?…. So, yeah,* ***I*** *think that [AHI test] would be great*. **Even if it took a little bit longer, and I had to hold them here in the clinic a little bit longer, if it tested sooner, that’s great, because you’re not always going to capture people to come back within 30 to 90 days.”** - Ana, Medical Assistant
Anxiety-reducing for *some*	*“if it’s someone who’s testing because of the specific experience, especially if it was non-consensual or something like that, they’ve already had to wait so long when* **why wait longer than necessary”** - *James, HIV and STI Tester*
**Limitations or Concerns** *	
Clients may be used to faster results	*“Um it’s hard though, because* **with the rapid tests we have now, that’s one of our gimmicks, you know, it’s like ‘it only takes a minute’**, *but I think as long as you work things in a way that make sense for your organization, then it’s it can definitely be worth it.” - James, HIV and STI Tester*
Organizational feasibility to accommodate wait time	*“It just, it depends. Like for our setting, there are a group of people who test and how we test,* **we try to, you know, see everyone within 30 to 45 minutes**. *So then, you know, our way will probably not be the best option for our site, but it may be the best option for someone else.” - Morgan, a Testing and Counseling Program Manager*

* only reported by staff

**Table 3:** Benefits and Concerns of End-User Type

Self-testing	Benefits	
	**Increases access/addresses barriers**	***“I think* self-testing, it’s got its place. *I think if they’re not going to come in here, because of the stigma, it’s great.” - Ana, Medical Assistant***
		***“I think especially with a lot of people who use IV drugs, it’s kind of an isolating lifestyle. And I think it becomes a barrier to healthcare having to… seek out resources for something… that there’s a giant social stigma for, and also, like, sometimes it’s just hard to find those programs.* Being able to do it at home would eliminate pretty much all obstacles.” - *Kiana, client***
	**Convenience**	***“I could do it… on my own schedule as opposed to going to a clinic” (Robert, client)***
	**Privacy** *	***“I think it’d be cool to be able to do it yourself, because a lot of times, I feel like some people might have that like sliver of doubt, to where, you know, they- if something did happen, maybe they would just want to be able to test themselves in private.” - Robert, client***
	**Peace of mind** *	***“I can be by myself and if I imagine any wonders or issues or whatever, then it’s just easy just do it and, you know, go on from there. Like I said, at least I know that I’m good on my end of it” - Natalie, client***
	**Limitations or Concerns**	
	**Accuracy** *	***“… like with pregnancy tests or with any other thing like that, there’s always that doubt of, well, you know, maybe it’s maybe it’s wrong, maybe I’m gonna have to go to a doctor to check and stuff” - Robert, client***
	**User ability to perform test correctly**	**“… *not everyone is great at like following directions… you might get an unclear result if you’re not following directions clearly” - Kiana, client***
	**User ability to interpret results** **	***“I just think that having somebody do a home test, they’re just not going to understand. You know, all they’re going to see is that that line, or those two lines, and they’re going to think they’re positive, and then being able to reach somebody. I mean if somebody tests on a Friday night at home, they would not be able to reach say our organizations until the following Monday because we’re not open on the weekend - Kennedy, Director of Outreach Services***
	**Self-reporting** **	***However, my concern is the self-reporting. If I’m self-testing, you’re home alone, you know, am I going to report myself if it does come out reactive? - Morgan, Testing and Counseling Program Manager***
	**Lack of pathway to follow-up treatment**	***I guess the biggest downside would be concerned about what action to take, willingness to take action?” - Jaime, client***
		***“… self testing is great but would they really let somebody know if they were positive, to be able to get into medical management, you know, treatment in that way. And you have to remember like we work a lot with people who use drugs, who inject. So, some of them might not be very forthcoming with that or just might not ever do anything about it. Sometimes it takes that extra push from us. You know it’s not a death sentence anymore, you can get better, we can do treatment, you know some of that encouragement. I don’t know that we would ever get them into care, that’s my biggest concern around here.” - Kayla, HIV Prevention Outreach Coordinator / Certified Peer Recovery Coach***
	**Lack of emotional support and counseling**	***“If a person turned out positive, they’re kind of all on their own at that moment. And that would be a traumatic moment… You don’t test yourself for cancer and find out all alone that you have cancer. So that would be dramatic.” - Terrence, client***
		***“ I think my main concern about that [self-testing] is especially with HIV and just because of those stigmas we talked about earlier, the counseling part after a positive test… there’s that support in an office of people [who] are educated in one, crisis intervention, and two, in HIV. I think is very important and if you test at home, you don’t have that component of it.” - Gia, Harm Reduction Program Manager***
	**Potentially exacerbating stigma**	***“But I think with advancing the move away from stigma and historical trauma of HIV from you know the 80s and the 90s, and the silencing and some of the public service campaigns that maybe dramatize HIV in not so helpful ways, I think that as we push to really get stigma dealt with, it’s not necessarily helpful to promote the idea that HIV testing is something that a person needs to do at home… it’s not quite that, it’s really not quite that sensitive.” - Anita, HIV Testing Department Manager***
**CHW/Peer-based testing**	**Benefits**	
	Addresses access barriers	*“You look at some of our counties we serve, they’re very very small, and they have no access to any testing whatsoever and if we didn’t go there, on a regular basis and do testing at a treatment center or at the county health department… They wouldn’t do it…* **you need to meet people where they’re at***.” - Kennedy, Director of Outreach Services*
	Skilled at building trust and rapport**	*“… as a community health worker you could get in there and really begin to understand what their needs are and what their wants are, and you could slowly bring in other people to kind of meet that [need]…to where it’s not just them coming to you… I think it can be a great way to combat HIV.” - Blaire, Director of Outreach and Testing*
	Can provide education, guidance and support	*“I think community health workers are helpful and they can help you understand a wide variety of things having to do with your health… somebody who can make sure that you understand what’s happening, right?” - Terrence, client*
	Unique bonds through shared life experiences	*“… we’ve had someone that had previous injection drug use and we tested and they were reactive. That same person [tester] was also living with HIV. The instant bond that they had and [how they] were able to communicate about it, their drug of choice that they both used, was an automatic bond… I have years of experience, but the rapport and how quickly they built that rapport with each other… and [to] have a really deep understanding of what the feelings that they were kind of going through even though they were different people, they had those similarities that were, I think it was really beneficial to that person.” - Blaire, Director of Outreach and Testing*
		*“it’s good when there’s someone, like I said, that knows kind of about it [drug use] because I hate people that they get hired at some of these places and then they haven’t, they’ve never lived it, they’ve never seen it and it’s hard to take somebody serious…” - Natalie, client*
	Can provide a safe, non- judgmental space**	*“It creates a sense of safety. The judgmental piece goes away, the awkwardness… I’m a person who used to inject drugs and so when I can talk about certain things and just like the way they talk about them and not the way, you know, like a medical person or a clinical person would talk about them. There’s, like, almost a sense of just like you can just feel them like loosen up and see them relax” - Gia, Harm Reduction Program Manager*
	**Limitations or *Concerns***	
	Limitations caused by budget and institutional policies**	*“Well, we are limited by our budget and we’re limited by what we as an institution can do… we used to go to the jails, and then they changed the rules… so now we can’t” - Andy, Outreach Coordinator and Tester, Community Health Worker*
	CHW/Peer capacity**	*“Our limitations are also based off of like you know what the worker is capable of doing with their time, like right now in our office there’s two of us. … I would say like rural communities are extremely difficult, because, like, we had a few sites that are like, an hour away. And that’s the general like limit that we can do because mileage, and how far we’re willing to drive on our time” - Andy Outreach Coordinator and Tester, Community Health Worker*
	Gaining community trust is hard work**	*“I think sometimes it’s seen as not as effective only simply because the people aren’t putting in the work to do it. They’re not putting in the work to understand the culture that they’re going into, and they become, they get rejected very quickly, and they just don’t put the effort in or they don’t have an understanding, or they communicate that no one’s at that venue. They just haven’t done the hard work” - Blaire, Director of Outreach and Testing*
	CHW/Peer safety and emotional wellbeing**	*“We also see occasionally somebody come through who hasn’t really dealt with their own baggage, so to speak… I’ve seen I think a lot of projection, I think a lot of countertransference, I’ve seen a lot of retraumatization happen in people, if they haven’t done that work. So yeah, absolutely peer based testing, hell yeah, but whoever’s managing that needs to be conscientious of the ways that that person that that staff member, that peer-based tester, can also get re-hurt, you know, or continually harmed in that process.” - Anita, HIV Testing Department Manager*

* only expressed by clients

** only expressed by staff

**Table 4:** Implementation Considerations for Each End-User Type

Self-testing	CHW/peer testing
1) **Ensure accuracy.** *“As long as I can trust the accuracy and, and, and all that of the test. “Yeah. Because, you know, why not get tested [laughs]? - Terrence, client*	1) **Provide training and education opportunities for CHW/peers without testing experience.** *“if we can train them and get them to read the results accurately, then I’m all for it” - Andy, Outreach Coordinator*
2) **Consider the cost to both clients and organizations and how that affects ability and willingness to adopt the test.** *“There’s also just a little frustration with the fact that I mean if you purchase one at CVS that are like $50, and you can come to us and get tested for free.” - Evy Prevention Team Lead*	2) **Hire people with experience and skills that foster communication and trust in communities.***”… we have to really befriend the communities that we want to get to know and earn their trust… finding those folks who can help build that relationship is really important.” - Evy, Prevention Team Lead*
3) **Create a test that is simple to use or “accident foolproof” with simple instructions.** *“I think as long as the instructions were clear and everything that would, that would be fine to me.” - Kiana, client*	3) **Incorporate the AHI test as an option for clients in addition to existing tests used by the organization.** *“Maybe [this test] can be something that a patient has a choice of [you tell them] ‘well this could find it [HIV] in two months versus this one finds within three months and the time periods…”” - Carissa, HIV and STI Tester*
4) **Include information on what to do if you test positive.** *“When these type of tests are going to be over the counter… they should also have some information about where people can go to do to do next, if they test positive.” - Jaime, client*	
5) **Find creative ways to achieve a “human element” to self-testing or to minimize loss to follow-up (e.g., online test assistance, education, emotional support/counseling, and/or healthcare navigation).** *“I would just hope that there was a human element, a way for someone to reach out to somebody that, just to help them deal with the information they received, you know.” - Blaire, Director of Outreach and Testing*	

## Data Availability

Data, coding schemes, and interview guides are available by request. Please email Natalia Rodriguez at natalia@purdue.edu.

## Abbreviations

AHI: acute HIV infection
CHW: community health worker
HIV: human immunodeficiency virus
POC: point-of-care
PWID: people who inject drugs
RT-LAMP: reverse-transcription loop mediated isothermal amplification
STI: sexually transmitted infection
